# Identifying meteorological factors influencing catechin biosynthesis and optimizing cultivation conditions of tea plant (*Camellia sinensis*)

**DOI:** 10.3389/fpls.2025.1532880

**Published:** 2025-02-20

**Authors:** Marat Tukhvatshin, Qiliang Peng, Xuan Zhao, Jianghong Liu, Ping Xiang, Jinke Lin

**Affiliations:** ^1^ College of Horticulture, Fujian Agriculture and Forestry University, Fuzhou, China; ^2^ College of Life and Environmental Science, Hunan University of Arts and Science, Changde, China

**Keywords:** tea, *Camellia sinensis*, meteorological factors, catechins, EGCG

## Abstract

Catechins, the most important bioactive components in tea plants (*Camellia sinensis*), are influenced by the growth environment. To identify and optimize the key meteorological factors affecting catechin accumulation, we investigated the relationship between meteorological factors and tea plant catechin biosynthesis across three growing seasons at 10 locations. Rainfall, average temperature, and effective accumulated temperature (EAT) were identified as key drivers regulating catechin accumulation via the responsive expression of key structural genes *CsCHS1*, *CsANR*, and *CsSCPL*. Optimal meteorological conditions for enhancing total esterified catechins (TEC) and total non-esterified catechins (TNEC) were determined using LINGO software, although the optimal conditions for these two groups were contrasting. Hot and rainy environments promote the biosynthesis of EGCG, ECG, and TEC through *CsPAL* and *CsSCPL*, while reduced rainfall and EAT promote the accumulation of C, EGC, and TNEC. This study reveals the differential effects of meteorological factors on catechin accumulation and obtains optimal meteorological conditions for promoting catechin accumulation. These results provide guidance for improving catechin accumulation and tea cultivation management.

## 1 Introduction

Catechins, a class of polyphenolic compounds predominantly found in tea plants (*Camellia sinensis*), have garnered significant attention for their health benefits, including antioxidant, anti-inflammatory, and cardioprotective properties (Higdon and Frei, 2003; Sinija and Mishra, 2008; Kaur et al., 2014). Among these catechins, epigallocatechin-3-gallate (EGCG) is considered one of the most potent due to its biological activity (Yang et al., 2009; Alemany-González et al., 2020; Tuo et al., 2024; Yu et al., 2024a). However, the biosynthesis of catechins is a highly complex process regulated by both genetic and environmental factors, with significant variability observed across different tea cultivars and growing conditions (Wei et al., 2011; Wang et al., 2021; Xiang et al., 2023). Catechin biosynthesis is mediated by a well-characterized set of enzymatic pathways, including the phenylpropanoid and flavonoid biosynthetic pathways (Lin et al., 2017; Gong et al., 2020; Xiang et al., 2021a, b). Key enzymes, such as chalcone synthase (*CsCHS1*), flavanone 3-hydroxylase (*CsF3H*), and leucoanthocyanidin reductase (*CsLAR*), have been identified as critical regulators of catechin production (Gong et al., 2020; Xiang et al., 2021a, b). However, while the genetic regulation of these enzymes is relatively well understood, the role of environmental factors in modulating their activity remains underexplored. Recent studies suggest that climatic conditions can significantly affect catechin profiles by altering gene expression and enzyme activity (Yao et al., 2005; Wei et al., 2011; Liu et al., 2015; Deka et al., 2021; Wang et al., 2021).

*Camellia sinensis* var. *sinensis* cv. *Tieguanyin*, commonly used to produce oolong tea also known as Tieguanyin or “Iron Goddess of Mercy,” is a globally recognized tea cultivar originating from Fujian Province, China (Guo et al., 2019; Chen et al., 2021; Zhang et al., 2021). This cultivar is distinguished by its unique flavor profile and aroma, primarily driven by the interplay of environmental factors, harvest season, and the resulting variation in secondary metabolite biosynthesis (Xu et al., 2018; Zhou et al., 2019a, b, 2025). The significant influence of these factors on biochemical composition underscores the importance of understanding their complex interactions to optimize cultivation practices and ensure consistent, high-quality tea production (Zhou et al., 2019a, 2025).

As global temperatures continue to rise due to climate change, the ability to optimize tea cultivation practices becomes increasingly important (Azapagic et al., 2016; Ahmed et al., 2018). Understanding the interaction between genetic and environmental factors in catechin biosynthesis will therefore be critical for ensuring the sustainability of tea production in the face of changing climatic conditions (Ahmed et al., 2018, 2019; Muoki et al., 2020). Optimization models that integrate genetic and environmental data could play a key role in guiding future cultivation practices (Tang et al., 2023; Wang et al., 2023; Zhu et al., 2024). By identifying the optimal temperature ranges and other environmental conditions for specific catechins, these models could help mitigate the adverse effects of climate change on tea quality.

Catechins are essential contributors to the quality of tea leaves. However, the ecological factors influencing catechin content are diverse and complex. Which ecological factors exert the greatest impact on catechin content? Under what ecological conditions do tea plants accumulate the highest levels of catechins? Addressing these questions allows tea growers to select cultivation sites based on ecological conditions that optimize catechin content in tea leaves. Moreover, in tea cultivation within plant factories, producers can apply the experimental findings of this study to establish specific environmental conditions, thereby artificially regulating catechin content. In this study, we collected data on eight meteorological factors across three seasons from 10 tea plantations in Anxi County, the origin of the *Tieguanyin* cultivar. These data were analyzed alongside catechin content and related gene expression levels. Partial least squares (PLS) analysis was employed to identify key meteorological factors influencing catechin biosynthesis, while LINGO was used to solve a multiple regression equation and determine the optimal meteorological environment for promoting catechin accumulation. These models could potentially inform breeding programs aimed at developing new tea cultivars that are more resilient to changing environmental conditions while maintaining desirable catechin profiles. This study specifically examined the relationship between various meteorological factors and catechin composition in tea plants across three growing seasons (spring, summer, and autumn) and 10 distinct geographical locations. The observed weather patterns varied significantly by location and season offering valuable insights into the environmental factors that influence catechin biosynthesis.

## 2 Materials and methods

### 2.1 Plant materials

Ten-year-old tea plants of the Tieguanyin cultivar (*Camellia sinensis* var. *sinensis* cv. *Tieguanyin*) were selected for experimental studies across 10 tea plantations. These plantations were managed under comparable agricultural practices, including similar pruning schedules, fertilization regimes, and pest control methods ensuring consistency in growth conditions across the experimental sites. Fresh leaf samples were collected during mid-April to late April for spring (2021), late July to mid-August for summer (2021), and late September to mid-October for autumn (2021). Samples were collected according to the “one bud and two leaves” standard. At each experimental site, sampling was conducted using a fixed quadrat frame (0.1 m²) to select designated sampling points. For each season, newly sprouted shoots were collected from Tieguanyin tea plants, with three biological replicates established per site. Each biological replicate consisted of 15 shoots resulting in a total of 45 shoots per site per season. For catechin detection, collected leaves were oven dried until completely dry and then stored at −20°C in the laboratory until further analysis. The oven was programmed for a two-step drying process as follows: 110°C for 10 min followed by 90°C for 20 min. Freshly collected leaf samples for freeze drying and following qPCR analysis were immediately immersed in liquid nitrogen for solidification and subsequently stored at −80°C in the refrigerator.

### 2.2 Overview of sampling sites

**Figure 1:**
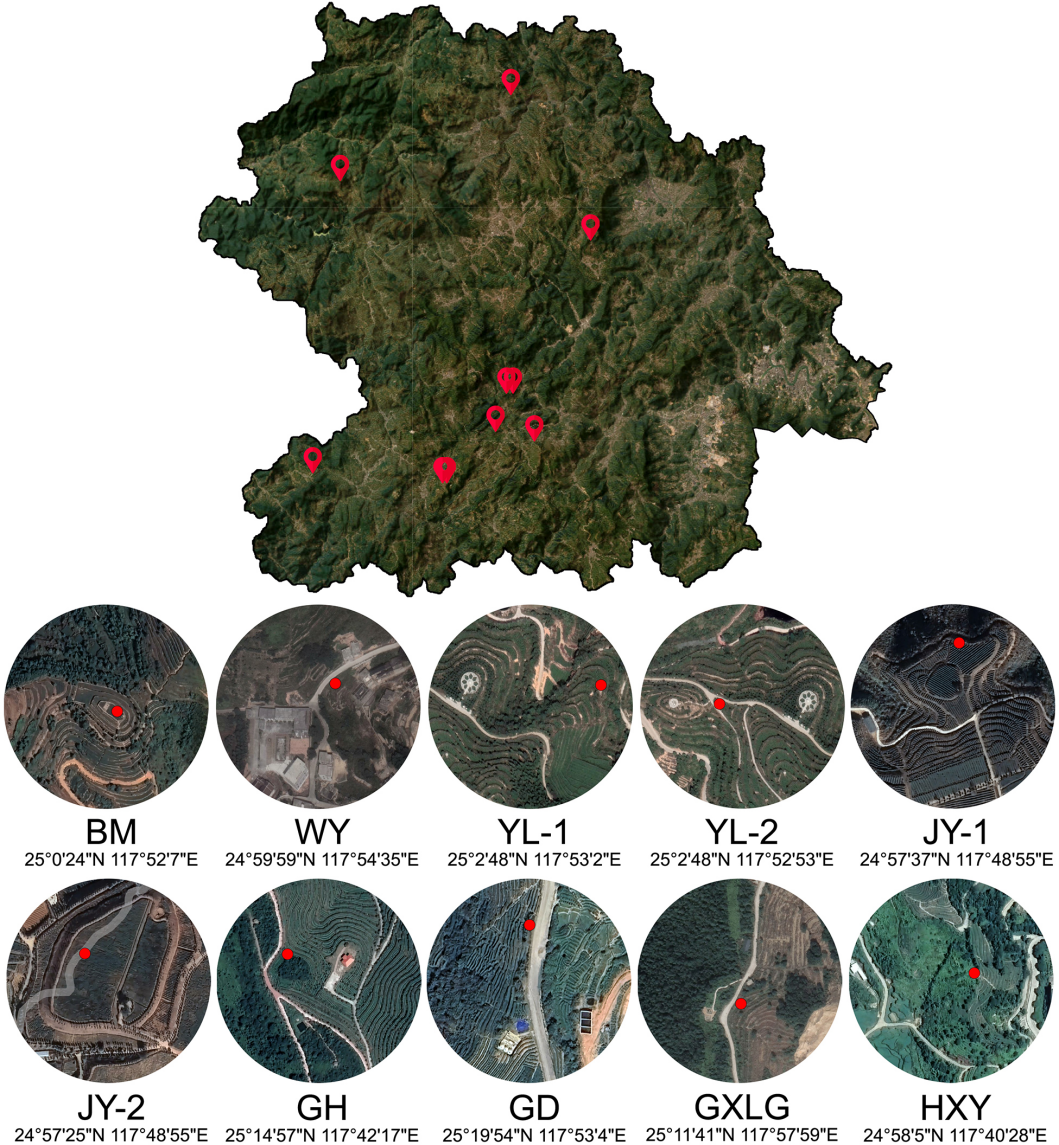
Overview of the sampling sites in Anxi county. This image shows a satellite map of Anxi County, with sample collection points and their geographical coordinates.

The study encompassed six major tea-producing townships in Anxi County, Quanzhou City, Fujian Province: Xiping, Longjuan, Gande, Xianghua, Lutian, and Shangqing. The tea plantations were located in Bama (BM), Weiyin (WY), Yunlin (YL-1 and YL-2), Juyuan (JY-1 and JY-2), Guanghe (GH), Guoxinlvgu (GXLG), Gande (GD), and Huaxiangyuan (HXY). Sampling was conducted across 10 ecological tea gardens within these townships comprising 10 distinct sampling points. Detailed information regarding the sampling locations is provided in **Figure 1**. The cultivation method for indoor samples was identical to that described by Xiang Ping et al (Xiang et al., 2023). The cultivation period was from June 1, 2021 to July 31, 2021. Parameters for temperature, light intensity, and substrate moisture are provided in **Supplementary Table 1**.

### 2.3 Meteorological data

Meteorological data were selected from 24-h, daily, and hourly records automatically collected by small weather stations located within nine sample tea gardens in the main tea-producing townships of Anxi County (YL-2 location do not have meteorological data). As pruning dates varied across different sampling sites, to ensure the accuracy of meteorological data, the start and end dates were determined based on the total number of days from the current season’s shoot pruning to the next season’s first round of new shoot germination reaching the “one bud and two leaves” stage. Specific meteorological factors selected included daily average rainfall, daily average temperature, effective accumulated temperature during the growing season (EAT), maximum temperature, minimum temperature, average relative humidity, average 10-cm ground temperature, and daily average irradiance for each sampling site. Meteorological data were recorded hourly using microclimate observation systems installed in the tea gardens and were provided by the Anxi County Meteorological Bureau, Quanzhou City, Fujian Province. Average daily temperature, 10-cm ground temperature, and humidity were calculated by averaging hourly measurements and subsequently computing daily averages over the entire seasonal observation period. Effective accumulated temperature (EAT) was determined using the formula:


EAT = Average daily temperature − Biological zero degree

For tea plants, the biological zero degree was set at +10°C. The EAT values were then averaged over the observation period. Maximum and minimum temperatures were derived from the highest and lowest temperature readings recorded during the observation period, respectively. The average daily rainfall was calculated by summing the rainfall on days with measurable precipitation and dividing it by the number of such days during the growing season, excluding days with no rainfall. For irradiance, the daily average photosynthetically active radiation (PAR) values were used, with extreme values excluded due to sensor malfunctions. The irradiance data were based on the PAR dose and maximum irradiance recorded ensuring accuracy by discarding faulty readings caused by equipment failures. Meteorological conditions are presented in **Supplementary Table 2**.

### 2.4 Catechin detection method

Tea samples (0.2 g, accurate to 0.0001 g) were weighed into a 10-ml centrifuge tube, and 5 ml of 70% methanol solution preheated in a water bath was added. After being shaken by a mixer, the tea was immediately transferred to a 70°C water bath. The tea was immersed for 10 min and shaken once at the 5-min mark, and the centrifuge tube was transferred to the centrifuge after 10 min (set to 3,500 r/min, for 10 min). The residue was extracted with an additional 5 ml of 70% methanol solution, and the procedure was repeated as described above. The combined extract volume was made up to 10 ml, shaken, and 1 ml of the extract was transferred to a 10-ml volumetric flask using a pipette, then diluted to 10 ml with a stabilizing solution and filtered through a 0.45-μM membrane before being analyzed by HPLC. The preparation method of the stabilizing solution is as follows: dissolve 25 ml of EDTA-2Na (concentration: 10 mg/ml), 0.2 g of ascorbic acid, and 50 ml of acetonitrile, and dilute the mixture with water to a final volume of 500 ml in a volumetric flask.

The HPLC instrument used was a Waters Acquity UPLC HSS T3 column (2.1 * 100 mm, RP 181.7 µm) set at a column temperature of 35°C. Mobile phase A consisted of 97.98% pure water + 0.02% EDTA-2Na + 2% glacial acetic acid, and mobile phase B consisted of 98% acetonitrile + 2% glacial acetic acid. PDA detection conditions were as follows: a scanning range of 200–400 nm, a characteristic detection wavelength of 278 nm, a scanning time of 10 min, and an injection volume of 2 μl. The standard stock solutions used are listed as follows: catechin stock solutions: C (1.00 mg/ml), EC (1.00 mg/ml), EGC (2.00 mg/ml), EGCG (2.00 mg/ml), and ECG (2.00 mg/ml). All samples were analyzed in three biological replicates, and mean values were calculated for data analysis.

Calibration curves were constructed using the injection concentration as the x-axis and the chromatographic peak area as the y-axis. Linear regression equations and correlation coefficients for the nine components are shown in **Supplementary Table 3**. After stabilizing the flow rate and column temperature, a blank run was performed (**Supplementary Figure 1**) to confirm a stable baseline with no extraneous peaks indicating good instrument performance. Then, the mixed standard sample was injected (**Supplementary Figure 2**) demonstrating good separation of individual catechin monomers. This study quantified three catechin categories: total esterified catechins (TEC), including EGCG, ECG, GCG, and CG; total non-esterified catechins (TNEC), including EGC, EC, and C; and total catechins (TC), representing the sum of all catechins (Liu-gan, 2014; Xiang et al., 2021a).

### 2.5 RNA extraction and qPCR

Total RNA was extracted from the samples using the RNAprep Pure Plant Kit (DP441; TIANGEN BIOTECH CO., LTD., Beijing, China) following the manufacturer’s instructions. First-strand cDNA was synthesized from the extracted RNA using the Script RT Kit (TIANGEN BIOTECH CO., LTD., Beijing, China) adhering to the manufacturer’s protocol. qRT-PCR was performed on an ABI 7500 Real-Time PCR System using the SuperReal PreMix Plus (SYBR Green) Kit (TIANGEN BIOTECH CO., LTD., Beijing, China) according to the manufacturer’s protocol. Each 20-µl reaction mixture contained 0.6 µl of forward and reverse primers, 1 µl of cDNA template, 10 µl of SuperReal PreMix Plus, and 7.8 µl of nuclease-free water. The PCR program consisted of an initial denaturation step at 95°C for 15 min, followed by 40 cycles of 95°C for 10 s and 61°C for 32 s. A melting curve analysis was performed at the end of the PCR cycles to verify the specificity of the amplified products (95°C for 15 s, 60°C for 1 min, 95°C for 30 s, and 60°C for 15 s). GAPDH was used as the internal reference gene for normalization. Relative expression levels of the target genes were calculated using the 2^−ΔΔCt^ method. Primer sequences used in the qRT-PCR analysis are listed in **Supplementary Table 4**. We diluted the cDNA 5, 25, and 125 times, respectively, to test the amplification efficiency (**Supplementary Figure 4**). The melting curve and amplification curve indicate the specificity of the primers (**Supplementary Materials 5**, **6**). Gene expression analysis was conducted by normalizing the expression levels of all samples to the BM plantation (used as the reference sample). Relative expression levels were calculated, and data were log2-transformed prior to visualization. Heatmaps were constructed using TBtools employing hierarchical clustering based on Euclidean distance to group both genes and samples.

### 2.6 Statistical analysis and optimization

Data organization and preliminary analysis were performed using Microsoft Excel 2019. Pearson’s correlation analysis, partial least squares (PLS) analysis, and regression analysis were conducted using SPSS software. Principal component analysis (PCA) was employed to investigate variations in catechin content among tea samples collected from 10 different plantations across three seasons. Partial least squares (PLS) analysis was performed to calculate the Variable Importance in Projection (VIP > 1) scores for meteorological factors influencing catechin content in Tieguanyin tea cultivar. Each catechin and gene expression level was treated as a dependent variable, while the eight selected meteorological factors (daily average rainfall, daily average temperature, effective accumulated temperature, maximum temperature, minimum temperature, average relative humidity, average 10-cm soil temperature, and daily average irradiance) were considered independent variables. Regression equations were constructed for each catechin based on the results revealing the relationships between meteorological factors and the concentration of individual catechins. root mean square error of calibration (RMSEC), root mean square error of cross-validation (RMSECV), and root mean square error of prediction (RMSEP) were computed to evaluate the performance of the partial least squares (PLS) regression models in Mathlab r2024a software. The optimization was conducted with LINGO 18.0 software. Using regression equation, the optimal values for each meteorological factor were calculated for maximizing the concentration of each catechin. As part of the model balancing process, catechins were divided into two groups: TEC and TNEC. When determining the maximum value and optimal conditions for a specific catechin, the regression equations for other catechins within the same group were also included in the model. However, threshold constraints were applied to their maximum and minimum values based on the data obtained in our study.

### 2.7 Graph build software

Cluster analysis and heatmap construction were performed using TBtools software. Statistical analysis and graphical representation of data were carried out using GraphPad Prism 10.0 and Adobe Photoshop software. Principal component analysis (PCA) was conducted and visualized using the online tools SRPlot and Chiplot (https://www.bioinformatics.com.cn/ and https://www.chiplot.online/, respectively). Optimization models were developed and analyzed using LINGO 18.0 software, and graphs were generated using GraphPad Prism 10.0 and Microsoft Excel 2019. Correlation maps and potential mechanism maps were constructed using Chiplot and Adobe Photoshop software.

## 3 Results

### 3.1 Significant seasonal and location-specific differences in catechin accumulation in tea plants

**Figure 2:**
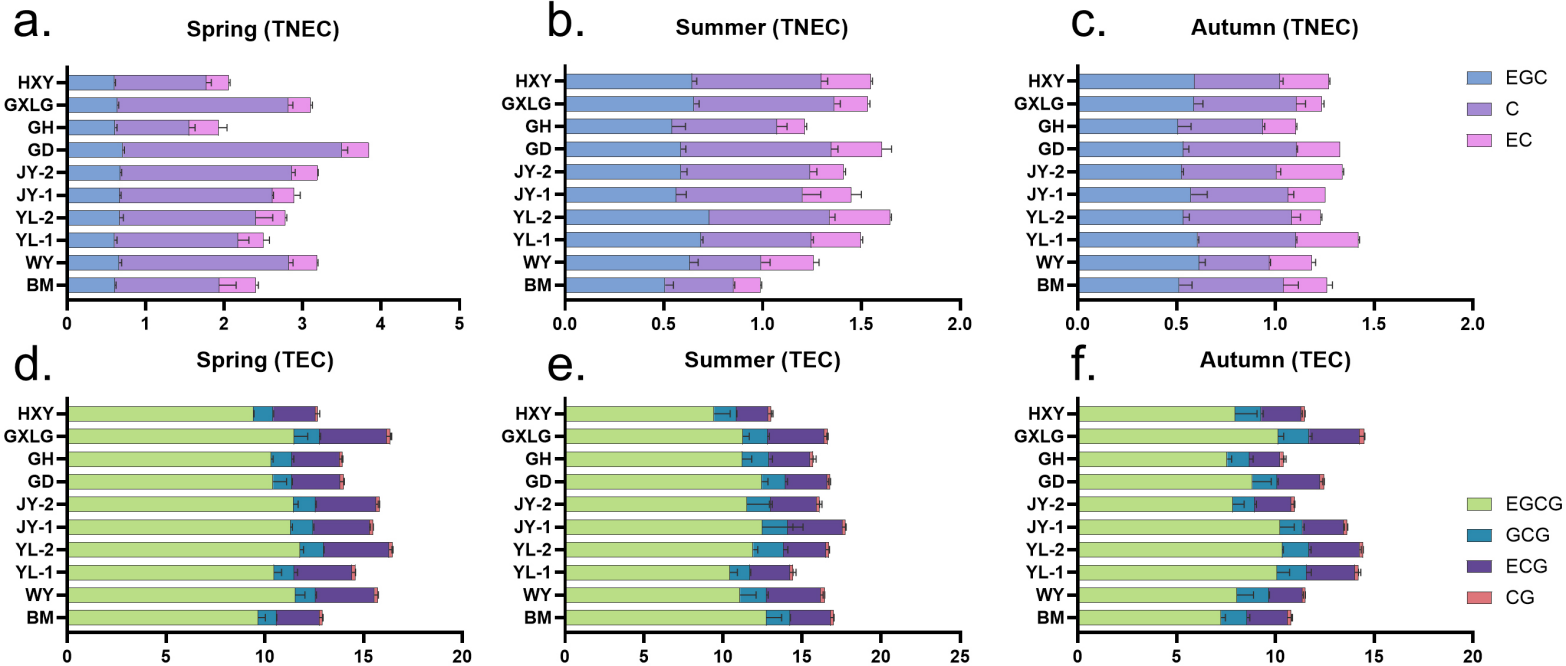
Influence of season and location on catechin content in tea plants. This graph shows the content of catechin across three seasons and 10 locations. TNEC is the abbreviation for total non-esterified catechins, and TEC is the abbreviation for total esterified catechins. **(a)** Content of TNEC in spring. **(b)** Content of TNEC in summer. **(c)** Content of TNEC in autumn. **(d)** Content of TEC in spring. **(e)** Content of TEC in summer. **(f)** Content of TEC in autumn.

The 10 locations showed significant differences in TNEC content across the three seasons (**Figure 2**). In spring, the highest TNEC content was found highest at GD with a value of 3.84%, largely due to the elevated C concentration (2.79%). In contrast, GH recorded the lowest TNEC (1.94%), with low values across all three catechins (**Figure 2A**). During the summer season, YL-2 and GD locations display the highest TNEC of 1.64% and 1.61% driven by the peak concentrations of EGC (0.73% and 0.59%) and C (0.61% and 0.73%) (**Figure 2B**). The lowest summer TNEC content was recorded in BM, where the total reached only 0.99%, with all non-esterified catechins showing a significant reduction, particularly C (0.35%) and EC (0.14%). In autumn, YL-1 exhibited the highest TNEC at 1.43% driven by elevated levels of EGC (0.61%) and EC (0.32%). On the other hand, GH recorded the lowest TNEC totaling 1.10% (**Figure 2C**). During the whole year observation, our findings suggest that the meteorological factors of the GD plantation are beneficial for TNEC accumulation, while those of GH are less favorable.

In spring, the highest esterified catechin (TEC) content was observed at YL-2 and GLXG, with a total of 16.49% and 16.35%, primarily due to the elevated concentrations of EGCG (11.79% and 11.48%) and ECG (3.32% and 3.38%) (**Figure 2D**). In contrast, HXY and BM recorded the lowest total content of 12.69% and 12.93%, with low values across all four catechins, particularly EGCG (9.41% and 9.65%) and CG (0.13% and 0.16%). During the summer season, JY-1 exhibited the highest TEC content, reaching a total of 17.74%, driven by EGCG (12.47%) and ECG (3.48%) (**Figure 2E**). The lowest content was observed at HXY, with a total of 13.05% mainly due to the reduced levels of EGCG (9.43%) and ECG (2.04%). In autumn, GLXG and YL-2 once again showed the highest esterified catechin content, totaling 14.48% and 14.43%, due to elevated concentrations of EGCG (10.14% and 10.35%) and ECG (2.55% and 2.59%). On the other hand, GH recorded the lowest content at 10.41%, with particularly low concentrations of EGCG (7.56%), ECG (1.58%), and CG (0.16%) (**Figure 2F**). Overall, the year-round observations suggest that the YL-2 and GLXG locations provide the most favorable conditions for the accumulation of TEC, while the conditions at HXY and GH are less conducive to their synthesis.

**Figure 3:**
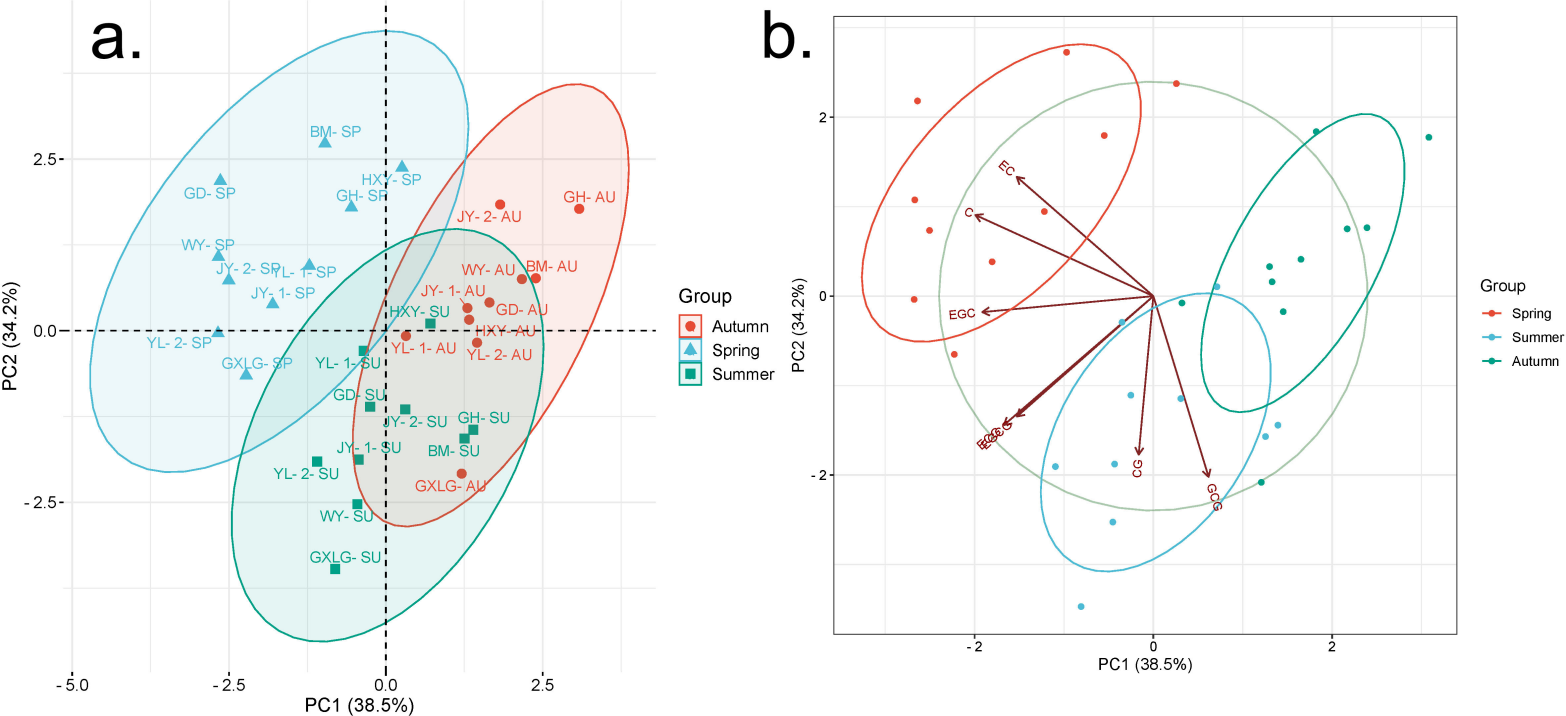
Principal component analysis of catechin content variation across three seasons. SP, spring; SU, summer; AU, autumn. **(A)** PCA plot; **(B)** PCA factor plot. This graph visually represents the results of a PCA analysis of catechin content across three seasons.

Principal component analysis (PCA) was used to examine the variations in catechin content among tea samples collected from 10 different plantations across three seasons (**Figure 3A**). The analysis showed that the first two principal components (PCs) explained 72.7% of the total variance, with standard deviations of 1.64 and 1.54, respectively. This indicates that seasonal changes in meteorological factors contribute to differences in catechin components. The loadings of individual catechins on these principal components reveal important patterns. EGC, C, EC, and EGCG all have strong negative loadings on PC1 suggesting a common response to seasonal factors among these catechins. The strong negative loading of C on PC1 (−0.5059) highlights its dominant role in explaining the variance across seasons. In contrast, GCG and catechin CG show higher loadings on PC2 indicating that these compounds may be influenced by different seasonal conditions compared to those more closely associated with PC1. Catechins, such as EGC, EGCG, and ECG, are particularly impacted by these environmental shifts, as reflected in their distribution across the principal components (**Figure 3B**).

**Figure 4:**
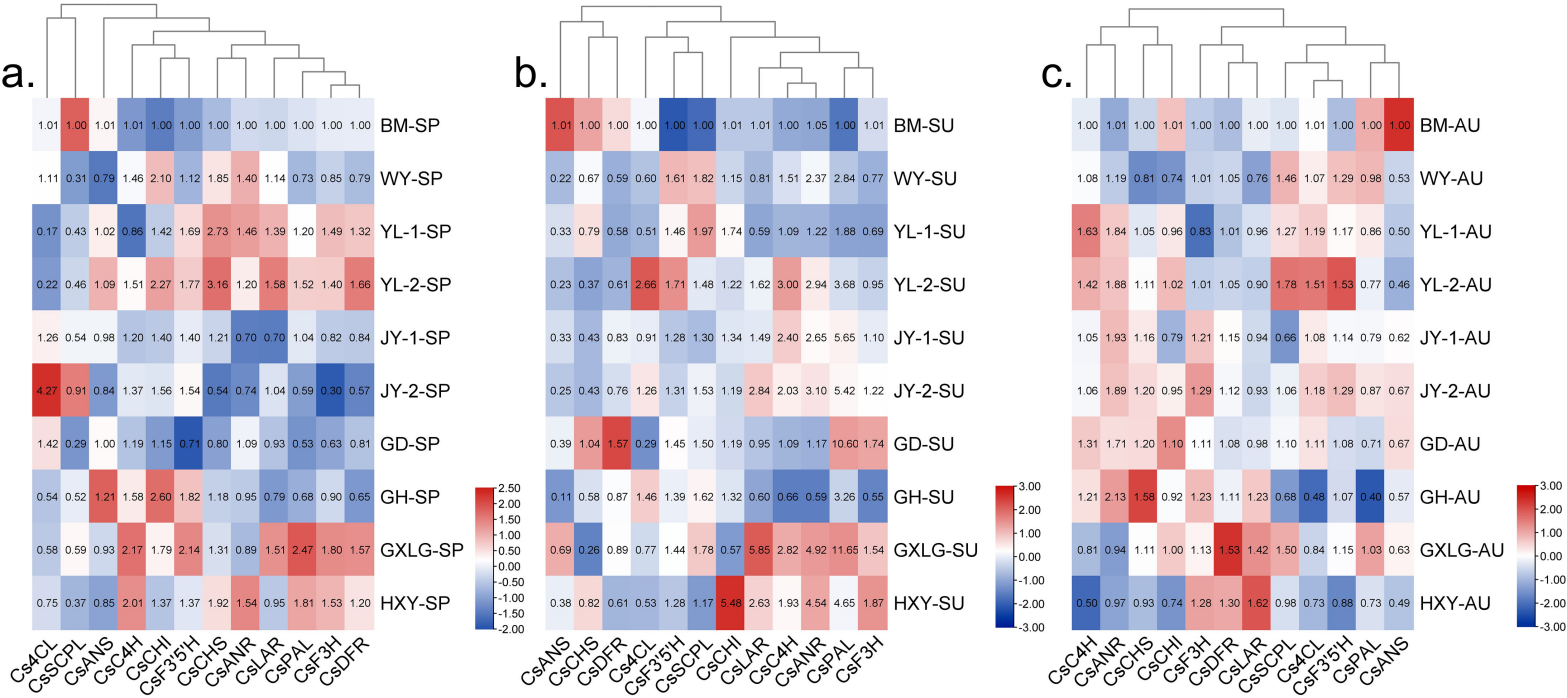
Heatmap of gene expression across three seasons and 10 locations (log2 transformed). SP, spring; SU, summer; AU, autumn. **(A)** Spring; **(B)** summer; **(C)** autumn. The heatmap shows gene expression across 10 locations and three seasons.

In spring, the expression of *CsPAL*, *CsF3H*, *CsDFR*, and *CsLAR* was upregulated at YL-1, YL-2, and GXLG influenced by meteorological factors observed at JY-2 and GD. (**Figure 4A**). During summer seasons, expression of *CsLAR*, *CsC4H*, *CsANR*, *CsPAL*, *CsF′3′H*, and *CsANS* genes at GLXG was upregulated, whereas at GH, WY, and YL-1, these genes was downregulated (**Figure 4B**). In autumn, seven genes (*CsANR*, *CsC4H*, *CsF3’5’H*, *Cs4CL*, *CsPAL*, *CsSCPL*, *CsCHI*) were upregulated at YL-1 and YL-2. In addition, the expression level of *CsF3H*, *CsDFR*, and *CsLAR* were found higher at GH, GLXG, and HXY, respectively (**Figure 4C**). During the whole-year observation, the GLXG location showed the most favorable meteorological factors for the expressions of *CsPAL*, *CsLAR*, and *CsDFR*.

### 3.2 Relationship between gene expression, catechin profiles, and meteorological factors in tea plants

**Figure 5:**
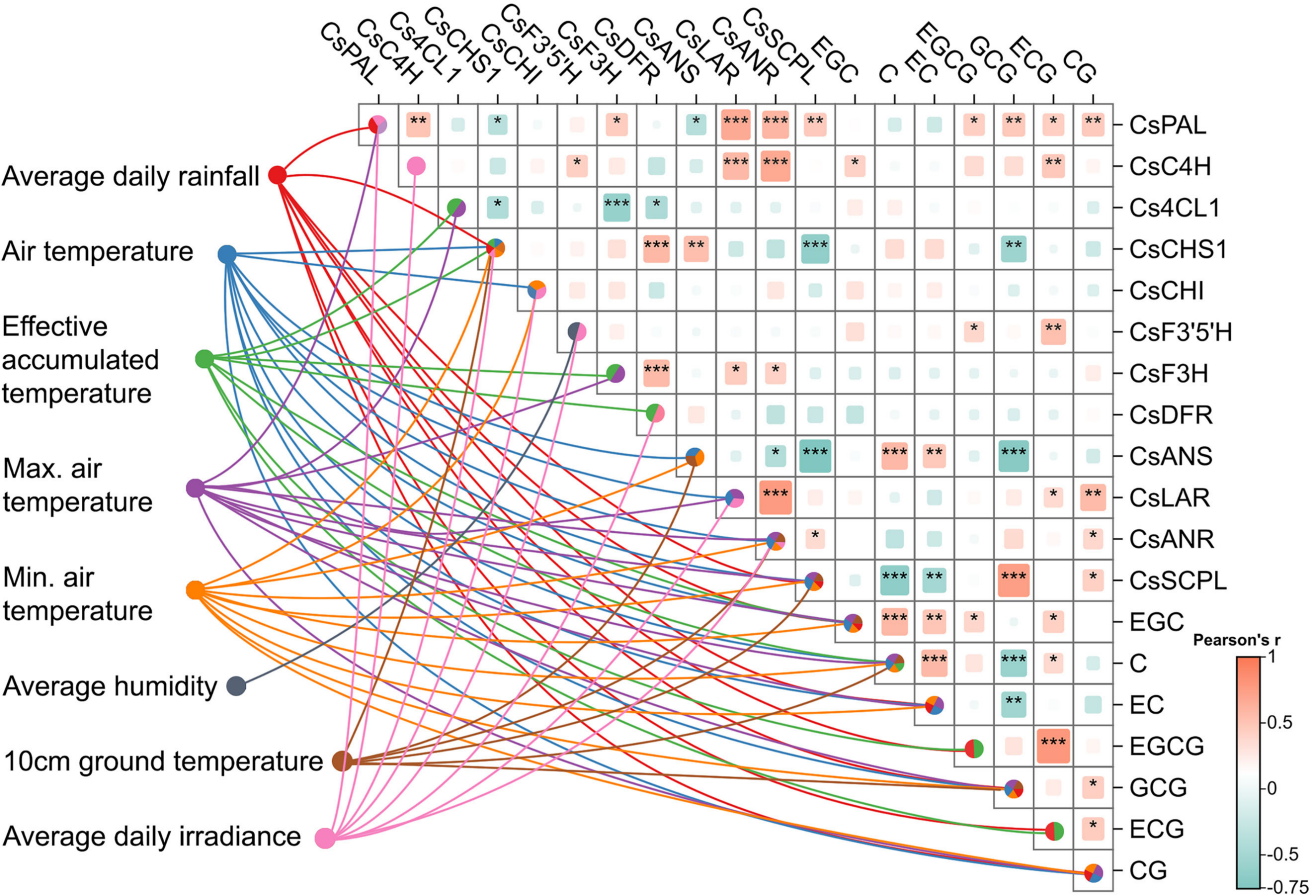
Relationship between catechin content, flavonoid biosynthesis gene expression, and the influence of meteorological factors. The lines represent the influence (VIP > 1) of meteorological factors (each with its own color) on gene expression and catechin synthesis. Intragroup correlation analysis and heatmap diagram shows Pearson correlation between gene expression and catechin content (*significance 0.01 < p ≤ 0.05, **0.001 < p ≤ 0.01, ***p ≤ 0.001). This graph illustrates the meteorological factors (VIP > 1) significantly influencing gene expression and catechin content. It also shows the correlation between different catechins and their corresponding genes.

To investigate the potential influence of meteorological factors on the biosynthesis of catechins and the expression of genes involved in their biosynthetic pathway, we performed a Pearson correlation analysis and calculated the Variable Importance in Projection (VIP) scores (**Figure 5**).

Meteorological factors exhibited a strong influence on the concentration of catechins in tea leaves. Notably, the concentration of non-esterified catechins, particularly EGC, was significantly affected by average daily rainfall, air temperature (average, maximum, and minimum), and EAT. C and EC displayed similar patterns of meteorological influence on their accumulation, with some variations. The content of EC was not found with high VIP value of EAT, while the content of C was not found with high VIP values of average daily rainfall, but was found with influence by 10-cm ground temperature (**Figure 5**). Maximum temperature, minimum temperature, and average temperature exhibited VIP values greater than 1 for the three non-esterified catechin monomers indicating that both average and threshold temperatures significantly influence non-esterified catechin content. Furthermore, average rainfall and EAT also significantly impacted non-esterified catechin monomer content. These findings suggest that the accumulation of non-esterified catechins is exceedingly influenced by various temperature factors and average daily rainfall.

Interestingly, the meteorological factors influencing the concentration of esterified catechins were the same as those affecting non-esterified catechins, but the impact on each individual catechin accumulation showed a different pattern. For instance, the content of EGCG and ECG was predominantly influenced by average daily rainfall and EAT. Notably, EAT demonstrated a VIP value exceeding 2 for ECG content. GCG and CG accumulation were influenced by average daily rainfall and air temperature (average, maximum, and minimum). Additionally, GCG accumulation was influenced by average 10-cm ground temperature (**Figure 5**). In conclusion, similar to non-esterified catechins, the biosynthesis of esterified catechins is primarily influenced by various temperature parameters and average daily rainfall.

The average, maximum, and minimum temperature values had VIP scores greater than 1 for the three non-esterified catechin monomers indicating a strong influence on the accumulation of non-esterified catechins. In contrast, average humidity and irradiance had a smaller effect on catechin content (VIP < 1). Average daily rainfall had a significant impact on the content of six catechin monomers, except for C. Among all factors, EAT exhibited the highest total VIP score across catechin accumulations. Both average rainfall and average temperature also displayed high cumulative VIP values underscoring their substantial influence on catechin accumulation. Overall, daily rainfall and various temperature factors, particularly EAT, significantly affect catechin accumulation in tea plants.

PLS analysis, integrating meteorological factors, gene expression, and catechin content, revealed a complex interplay between environmental conditions and the regulation of catechin biosynthesis in tea plants (VIP > 1). Average daily irradiance emerged as a crucial driver significantly influencing the expression of several genes in the catechin biosynthetic pathway, including *CsPAL*, *CsC4H*, *CsCHS1*, *CsCHI*, *CsF3’5’H*, *CsDFR*, *CsLAR*, and *CsANR* (**Figure 5**). This highlights the importance of light availability in stimulating catechin-related gene expression. Higher rainfall levels were also linked to the expression of *CsPAL*, *CsCHS1*, and *CsSCPL* indicating its role in modulating gene expression within the phenylpropanoid pathway and impacting catechin conversion.

EAT significantly influenced *CsC4H*, *Cs4CL1*, *CsF3H*, and *CsDFR* suggesting its involvement in regulating flavonoid pathway and the phenylpropanoid pathway. The average air temperature primarily affected *CsCHS1*, *CsCHI*, *CsANS*, *CsLAR*, *CsANR*, and *CsSCPL*, while minimum air temperature impacted *CsCHS1*, *CsCHI*, *CsANS*, *CsANR*, and *CsSCPL.* The maximum air temperature regulated *CsPAL*, *Cs4CL1*, *CsF3H*, *CsLAR*, *CsANR*, and *CsSCPL* indicating that cumulative heat exposure plays a key role in the transcriptional regulation of the catechin biosynthetic pathway. In summary, the average air temperature, minimum air temperature, maximum air temperature were identified as having important effects on *CsANR* and *CsSCPL* expression indicating that both the average and threshold values of temperature are crucial for the activation of the flavonoid pathway and its downstream regulation, particularly in the synthesis of galloylated catechins.

### 3.3 Optimization scheme of meteorological factors to improve catechins content in tea plants

The regression models reveal the relationship between meteorological factors and the concentration of each catechin (**Table 1**). The RMSEP, RMSECV, and RMSEC values were calculated to evaluate the performance of the model. RMSEP represents the error in predictions on the test set, RMSECV reflects the error from k-fold cross-validation, and RMSEC measures the calibration error on the training set. In general, RMSEC values were slightly higher than RMSEP and RMSECV, suggesting that the model was better at predicting the test and validation data compared to the training data. These results indicate that the model’s predictions are relatively accurate, with minimal overfitting observed in most cases. To assess the influence of meteorological factors on catechin content in young tea plant leaves, a system of linear programming equations was developed. These equations utilized the minimum and maximum values of meteorological factors obtained from each sampling point across the tea plantations. Based on VIP values and weights, optimal values for each meteorological factor were calculated using LINGO software to ensure the maximum concentration of each catechin.

**Table 1:** Regression models for predicting catechin levels based on meteorological factors.

Catechins	Regression equation	RMSEP	RMSECV	RMSEC
EGC	y = 0.14 − 0.009 * x_1_ − 0.002 * x_2_ − 0.0002 * x_3_ + 0.012 * x_4_ − 0.002 * x_5_ + 0.003 * x_6_ + 0.004 * x_7_ + 0.000004 * x_8_	0.00042	0.00044	0.00044
C	y = −1.08 − 0.054 * x_1_ + 0.122 * x_2_ − 0.002 * x_3_ − 0.006 * x_4_ − 0.077 * x_5_ + 0.038 * x_6_ − 0.048 * x_7_ − 0.000003 * x_8_	0.00229	0.00242	0.00538
EC	y = 1.538 − 0.002 * x_1_ − 0.006 * x_2_ + 0.0001 * x_3_ − 0.0029 * x_4_ − 0.001 * x_5_ − 0.005 * x_6_ + 0.005 * x_7_ + 0.000003 * x_8_	0.00049	0.00057	0.00051
EGCG	y = 8.637 + 0.367 * x1 − 0.042 * x2 − 0.002 * x3 + 0.079 * x4 − 0.031 * x5 + 0.024 * x6 − 0.087 * x7 − 0.00001 * x8	0.01184	0.01329	0.01559
GCG	y = −0.633 + 0.031 * x_1_ + 0.011 * x_2_ − 0.0004 * x_3_ + 0.037 * x_4_ + 0.002 * x_5_ + 0.005 * x_6_ + 0.009 * x_7_ − 0.00004 * x_8_	0.00105	0.00120	0.00206
ECG	y = 1.27 + 0.094 * x_1_ + 0.06 * x_2_ − 0.002 * x_3_ + 0.078 * x_4_ − 0.01 * x_5_ + 0.001 * x_6_ − 0.012 * x_7_ − 0.00004 * x_8_	0.00325	0.00363	0.00847
CG	y = −0.122 + 0.0005 * x_1_ + 0.001 * x_2_ − 0.00004 * x_3_ + 0.008 * x_4_ − 0.0003 * x_5_ + 0.001 * x_6_ − 0.0005 * x_7_ − 0.000002 * x_8_	0.00020	0.00021	0.00038

The meteorological factors (denoted as “x”) in both the regression and optimization equations include the following: average daily rainfall (x1), air temperature (x2), EAT (x3), maximum air temperature (x4), minimum air temperature (x5), average humidity (x6), average 10-cm ground temperature (x7), and average daily irradiance (x8).

**Figure 6:**
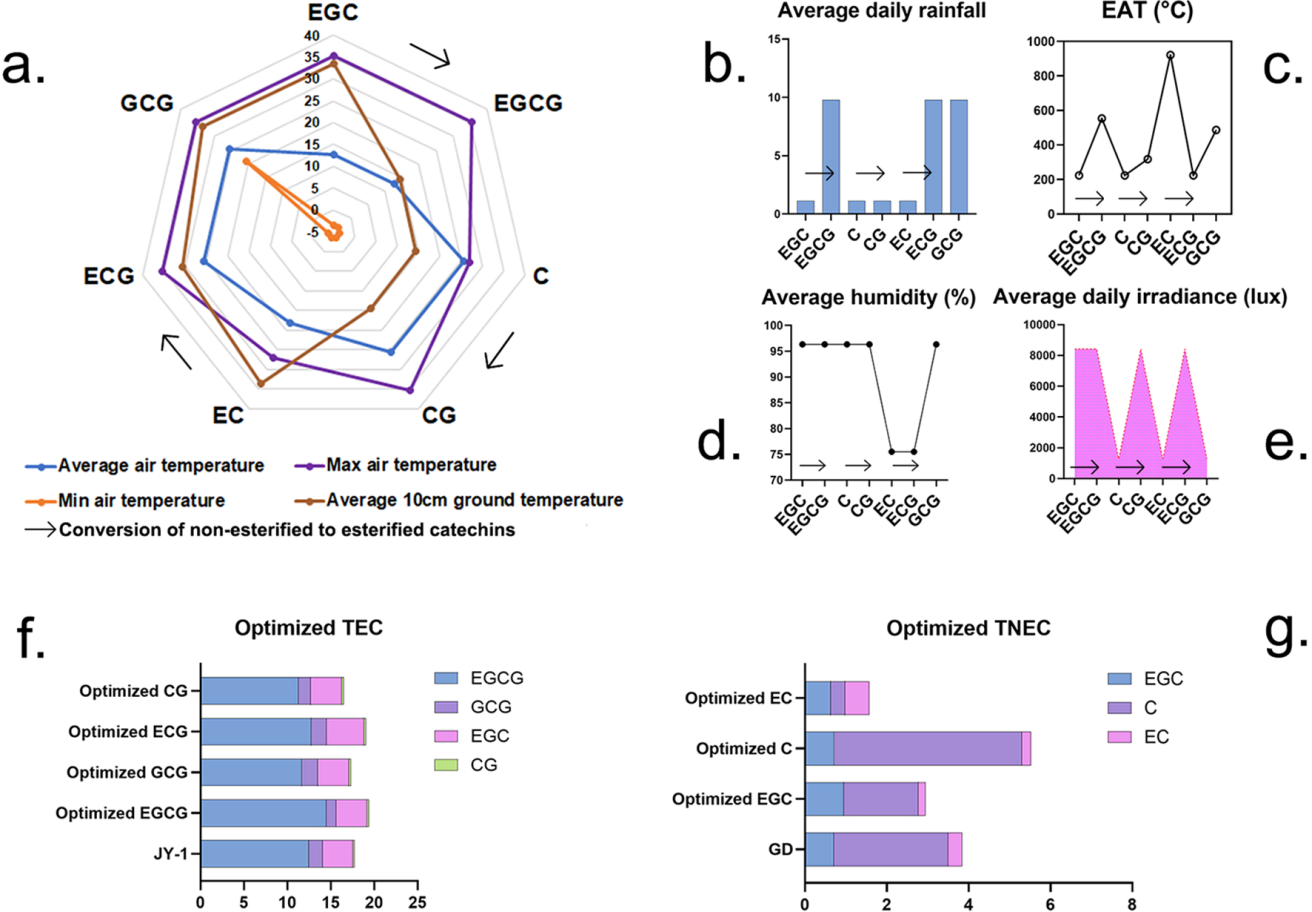
Optimized meteorological conditions and corresponding optimized catechin levels. **(A)** Various temperature factors, **(B)** average daily rainfall, **(C)** EAT, **(D)** average humidity, **(E)** average daily irradiance, **(F)** optimized and highest observed TEC value, **(G)** optimized and highest observed TNEC value. Description: These graphs show the optimal values for the synthesis of various catechins. Individual graphs represent specific meteorological factors. Graphs **(F, G)** show the predicted catechin content using optimization models, along with the maximum observed value.

For EGCG, under optimal meteorological conditions (9.78 mm rainfall, 12.72°C average air temperature, 553.74 EAT, 35.30°C maximum air temperature, −3.60°C minimum air temperature, 96.34% average humidity, 14.36°C 10-cm ground temperature, and 8,421.08 lux irradiance), the concentration reached 14.46% (**Figures 6A–F**). This represents a notable increase of 13.49% compared to the previously observed highest concentration of 12.74%. On the other hand, during optimization of ECG, under different meteorological conditions, such as 9.78-mm rainfall, 25.53°C average air temperature, 223.50 EAT, 35.30°C maximum temperature, −3.60°C minimum temperature, 75.50% average humidity, 30.65°C 10-cm ground temperature, and 1,283.50 lux irradiance, high values of ECG and TEC were also observed. The optimization scheme for these two catechins could improve the concentration of TEC reaching over 19% (**Figure 6F**). These data suggest that increased rainfall and elevated maximum temperatures promote the accumulation of EGCG, ECG, and TEC.

During the optimization of different TNEC catechins, only the optimization scheme for catechin C improved TNEC content. Optimized meteorological factors were as follows: 1.13-mm rainfall, 25.53°C air temperature, 223.50 EAT, 26.9°C maximum air temperature, −3.6°C minimum temperature, 96.34% average humidity, 14.36°C 10-cm ground temperature, and 1,283.50 lux irradiance. Under these optimized environmental conditions, C content reached 4.58%, and TNEC content was 5.52%, representing an increase of 63.8% and 43.75%, respectively, compared to the observed maximum values over the study period. This suggests that reduced rainfall, lower maximum temperatures, and decreased EAT contribute to higher TNEC accumulation (**Figures 6A–D, G**). In summary, higher daily rainfall and maximum air temperature could improve EGCG and TEC accumulation, while reduced rainfall and decreased EAT contribute to higher TNEC accumulation.

We established two distinct cultivation environments in the artificial climate chamber (T1: 25°C, 350 μmol·m−²·s−¹, 85% RH; T2: 20°C, 250 μmol·m−²·s−¹, 75% RH) and measured catechin content (**Supplementary Table 1**). Compared to T2, the T1 treatment resulted in increases of 28.69%, 26.03%, and 13.94% in EGCG, TEC, and TC levels in tea plants, respectively. This indicates that max temperature, light intensity, and humidity promote the accumulation of esterified catechins in tea plants. Conversely, under the T2 treatment, the total non-esterified catechins increased by 38.96%. Therefore, the catechin content of tea plant samples cultivated indoors aligns with the trends predicted by the optimized model: increased rainfall and higher temperatures promote TEC accumulation, whereas reduced rainfall and lower temperatures lead to increased TNEC accumulation.

### 3.4 Potential pathways through which meteorological factors influence catechin biosynthesis in tea plants

**Figure 7:**
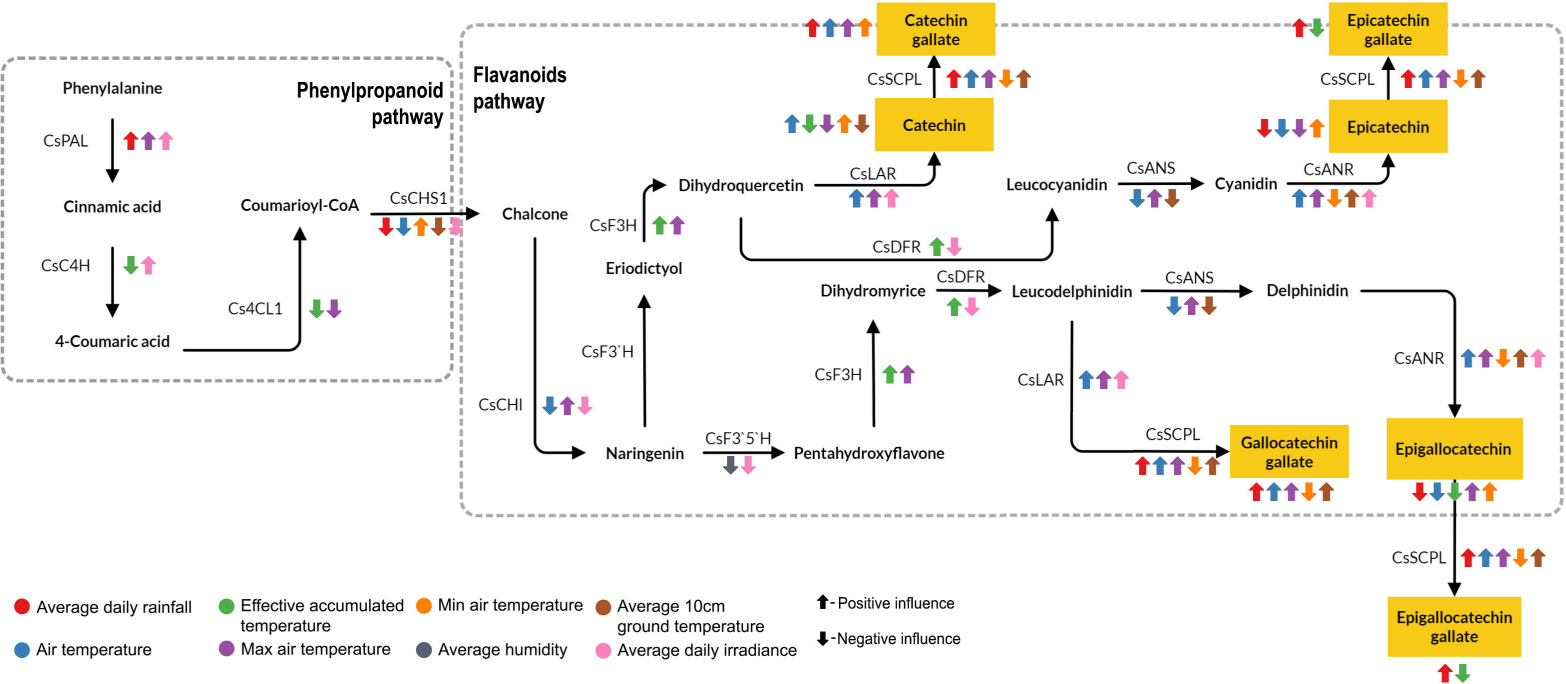
Potential mechanism of meteorological factors influences catechin biosynthesis in tea plants. The figure depicts the phenylpropanoid and flavonoid pathways of catechin biosynthesis indicating the genes involved at different stages. Colored arrows illustrate the influence of specific factors on the expression of relevant genes.

Meteorological factors exert a strong influence on the catechin biosynthetic pathway. C content was negatively influenced by EAT through the expression of *CsC4H*, *Cs4CL*, *CsF3H*. Also, the C pathway was affected by average daily irradiance that influences expression of *CsPAL*, *CsC4H*, *CsCHS1*, *CsCHI*, and *CsLAR*. Average rainfall, average temperature, and maximum temperature convert C to CG through the expression of *CsSCPL*. In addition, EC content is affected by average air temperature through the regulation of the genes involved in the flavonoid pathway, such as *CsCHI*, *CsLAR*, *CsANS*, and *CsANR*. Then, the high rainfall promoted C and EC conversion to CG and ECG through the regulation of *CsSCPL* (**Figure 7**). Average daily rainfall and EAT significantly impact the initial synthesis of phenylpropanoids and influence the expression of *CsPAL* and *CsC4H*. Within the flavonoid pathway, EAT influences the expression of *CsF3H* and *CsDFR*, while average daily rainfall impacts the later stages affecting *CsSCPL* expression and the formation of galloylated catechins. Furthermore, rainfall appears to promote the conversion of EGC to EGCG mediated by *CsSCPL* activity.

## 4 Discussion

### 4.1 Temperature is a key meteorological factor affecting tea plant biosynthesis

The sensitivity of tea plant metabolism to environmental conditions, particularly temperature fluctuations, has been well documented (Wang et al., 2011; Ahmed et al., 2019). The importance of temperature in modulating catechin profiles throughout the year has been revealed (Zhou et al., 2019a; Deka et al., 2021). In this study, the effective accumulated temperature (EAT) showed a strong correlation with catechin concentrations highlighting the importance of temperature for the production of these bioactive compounds (Wei et al., 2011). Interestingly, minimum and maximum temperatures also significantly influenced catechin concentrations suggesting that extreme temperature conditions play a particularly important role in regulating their synthesis (Wang et al., 2022; Zhang et al., 2023). Temperature (maximum, minimum, average, and accumulated) significantly affected the accumulation of non-esterified catechins, including EGC, C, and EC, consistent with previous findings (Yao et al., 2005; Huang et al., 2022). Our results suggest that temperature has a greater impact on non-esterified catechins than on esterified catechins. This observation aligns with our previous research, which demonstrated opposing response patterns to different temperature treatments (Xiang et al., 2023a). Previous studies have also reported contrasting accumulation patterns of esterified and non-esterified catechins under different seasons (Liu et al., 2015; Paiva et al., 2021).

Temperature modulates catechin biosynthesis through transcriptional regulation and enzymatic activity, particularly involving genes responsive to temperature changes (Zhao et al., 2023; Zhu et al., 2023). Our findings indicate that maximum, minimum, and average temperatures significantly influence the expression of downstream genes involved in catechin biosynthesis (CsLAR, *CsANR*, and *CsSCPL*). Previous research has also demonstrated that high or low temperatures affect the expression of *CsLAR* and *CsANR*, thereby altering catechin biosynthesis in tea plants (Zheng et al., 2016; Shen et al., 2019). The increased expression of *CsSCPL* during warmer periods aligns with enhanced accumulation of galloylated catechins (Chiu et al., 2016; Sun et al., 2018; Ahmad et al., 2020). This suggests that under heat stress, metabolic resources may be redirected away from the biosynthesis of lignin and flavonoids potentially prioritizing the synthesis of protective flavonoids (Lin et al., 2021; Teng et al., 2021; Li et al., 2022). However, further experimental evidence is required to confirm this redirection in metabolic resources, which would prioritize temperature-responsive pathways, such as those regulated by *CsSCPL*, over light-responsive ones (Zhang et al., 2017; Yang et al., 2022; Yu et al., 2024b).

Overall, the data strongly indicate that temperature is the most significant meteorological factor influencing the synthesis of both esterified and non-esterified catechins in tea plants. These findings have important implications for understanding how climate variations, particularly temperature changes, can affect the production of bioactive compounds in tea and other thermally sensitive crops.

### 4.2 Optimal ecological conditions for promoting catechin accumulation in tea plants

Our optimization model indicates that TNEC accumulation is predominantly driven by specific environmental conditions: low rainfall, low EAT, as well as low maximum and minimum temperatures, coupled with low irradiance. In contrast, the highest TEC accumulation was observed under conditions of high rainfall, elevated temperatures, and strong irradiance, primarily due to the highest content of EGCG. In summer, TEC content increases, which is related to increased temperature and rainfall (Bokuchava and Skobeleva, 1969; Yao et al., 2005; Deka et al., 2021). *CsSCPL* has been identified as a key enzyme promoting the conversion of EGC to EGCG, as supported by previous studies (Ahmad et al., 2020; Yao et al., 2022). Studies have shown that *CsSCPL* expression is modulated by temperature and also responds to drought stress (Chiu et al., 2016; Gong et al., 2020). Our research further reveals that temperature and rainfall significantly influence *CsSCPL* expression indicating that elevated temperatures and rainfall enhance the conversion of TNEC to TEC through *SCPL* regulation. Future studies are warranted to further validate these observations.

Based on our results showing a significant increase in EGCG content under drip irrigation during warm and dry periods, we recommend implementing drip irrigation to maintain optimal moisture levels and maximize TEC production, particularly EGCG. Selecting planting regions with average temperature ranges between 12.7°C and 25.5°C and effective accumulated temperature (EAT) within 220°C–550°C, coupled with high light intensity, can promote the synthesis of esterified catechins. Cooler and drier regions favor the accumulation of TNEC. Furthermore, considering the accumulation of non-esterified catechins during dry periods, farmers can strategically select regions with suitable temperature regimes and EAT to enhance the synthesis of esterified catechins. To mitigate excessive light exposure and stabilize temperatures in extreme climates, we recommend employing protective technologies such as shade nets (Saijo, 1980; Ji et al., 2018; Ye et al., 2021).

### 4.3 Meteorological factors influence catechin accumulation by regulating the metabolic flux within the biosynthetic pathway

Our results reveal that the phenylpropanoid pathway, primarily driven by *CsPAL* and *CsC4H*, responds more robustly to irradiance and temperature. This observation is consistent with previous studies demonstrating that light intensity significantly regulates the expression of genes involved in catechin biosynthesis within the phenylpropanoid pathway, including *CsPAL* and *CsC4H* (Teng et al., 2021; Chen et al., 2022). High maximum temperatures were linked to increased expression of *CsPAL*, *CsANR*, *CsLAR*, and *CsSCPL* promoting the accumulation of several catechins, while simultaneously potentially driving the conversion of EC to EGCG through increased *CsSCPL* activity (Li et al., 2020; Xiang et al., 2021a; Zhao et al., 2022). Low minimum temperature favored *CsCHS1* expression, while suppressing *CsSCPL*, influencing the accumulation of EGC, EC, C, and CG (Li et al., 2023; Zhao et al., 2024; Yu et al., 2025).

Rainfall can influence the late stages of catechin biosynthesis, particularly galloylation, by activating *CsSCPL*, which is involved in the conversion of catechins to their galloylated forms (Chen et al., 2024). Thus, the favorable conditions created by rainfall can enhance the accumulation of galloylated catechins, such as EGCG. A previous study shows that during monsoon season, a high level of TEC is observed, while drought can significantly decrease TEC content (Deka et al., 2021; Lv et al., 2021). This activation likely depends on sufficient hydration and nutrient availability, but experimental confirmation of *CsSCPL* functional dependency on moisture levels is needed.

While our study focuses on meteorological factors, such as temperature, light intensity, and rainfall, other factors like soil conditions and the presence of specific microorganisms may also influence catechin biosynthesis (Yang et al., 2018; Ahmed et al., 2019). Future research should consider these variables to gain a more comprehensive understanding of the regulation of these biosynthetic pathways.

## 5 Conclusion

Average rainfall, average temperature, and EAT are key meteorological factors affecting catechin accumulation in tea plants. Furthermore, the optimal meteorological conditions promoting esterified and non-esterified catechin accumulation are contrasting, due to the regulation of conversion by *CsSCPL*. Hot and rainy environment promoted TEC accumulation, while the decreased rainfall level and EAT were optimal conditions for enhancing TNEC. These findings provide valuable insights into the interaction between tea plant catechin metabolism and the environment resulting in optimized meteorological conditions for promoting catechin accumulation and informing cultivation.

## Data Availability

The original contributions presented in the study are included in the article/**Supplementary Material**. Further inquiries can be directed to the corresponding authors.
