# Supplementary material for: Identifying meteorological factors influencing catechin biosynthesis and optimizing cultivation conditions of tea plant (Camellia sinensis)
Source: Front Plant Sci. 2025 Feb 20;16:1532880. doi: 10.3389/fpls.2025.1532880 (PMC11882562; doi:10.3389/fpls.2025.1532880)
Supplement: Supplementary file 1 [file DataSheet1.docx]

Supplementary Material

# Supplementary Figures

#
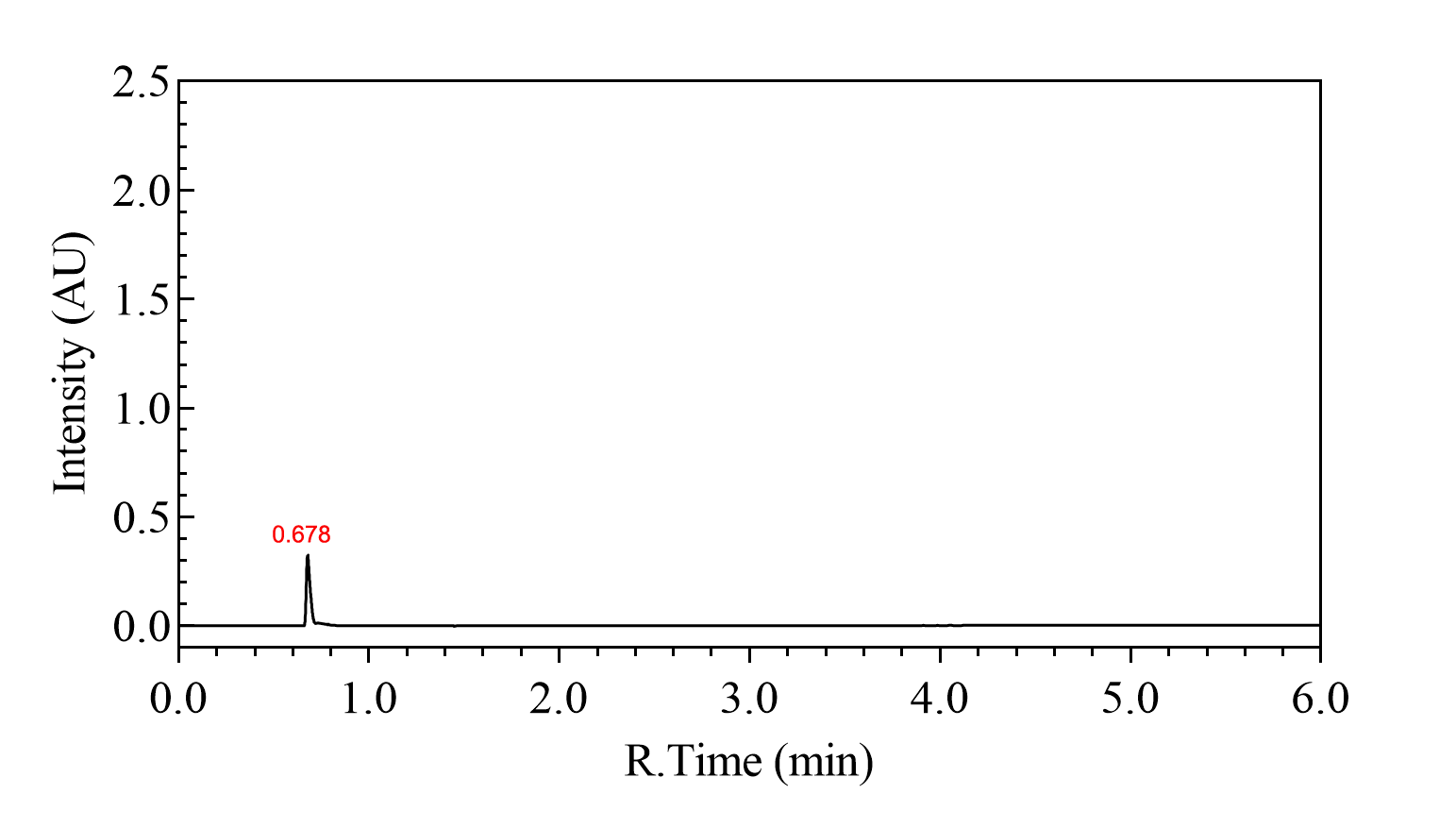


**Supplementary Figure 1.** Blank chromatogram.
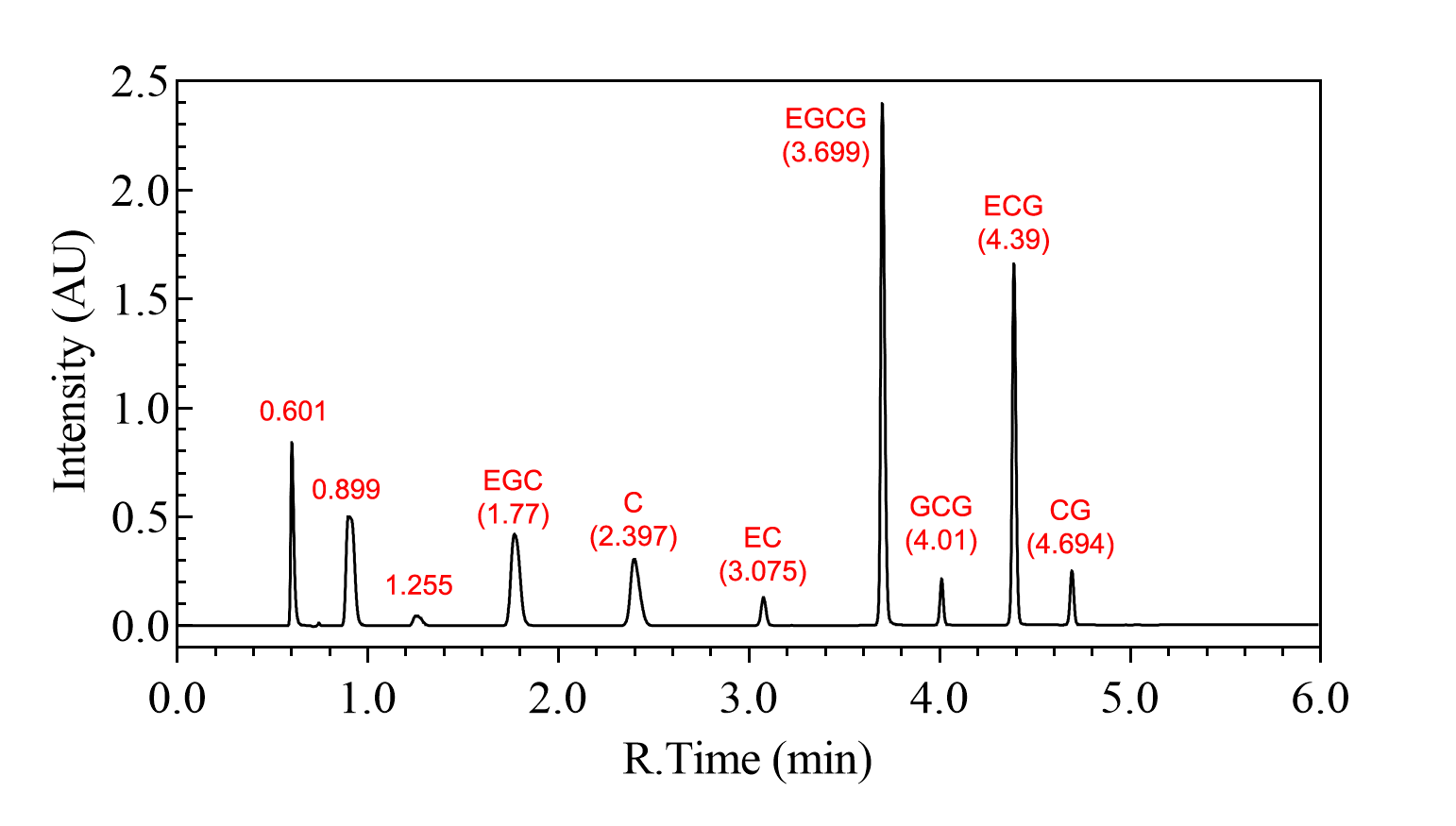


# Supplementary Figure 2. Peak time of each standard sample of the mixed solution. Epigallocatechin (EGC), catechin (C), epicatechin (EC), epigallocatechin gallate (EGCG), gallocatechin gallate (GCG), epicatechin gallate (ECG) and catechin gallate (CG).

**
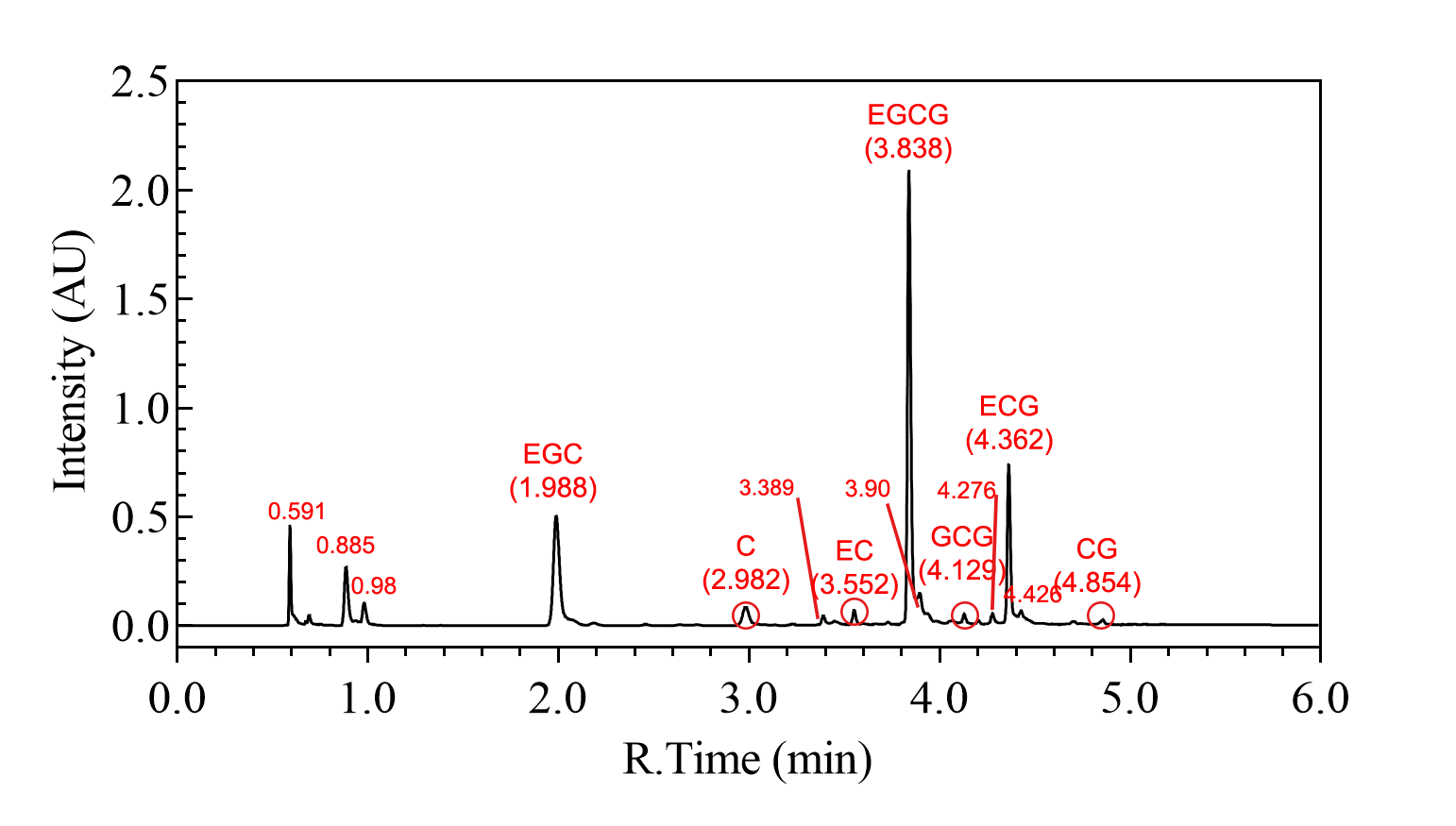
**

**Supplementary Figure 3.** Chromatogram of tea sample. Epigallocatechin (EGC), catechin (C), epicatechin (EC), epigallocatechin gallate (EGCG), gallocatechin gallate (GCG), epicatechin gallate (ECG) and catechin gallate (CG).


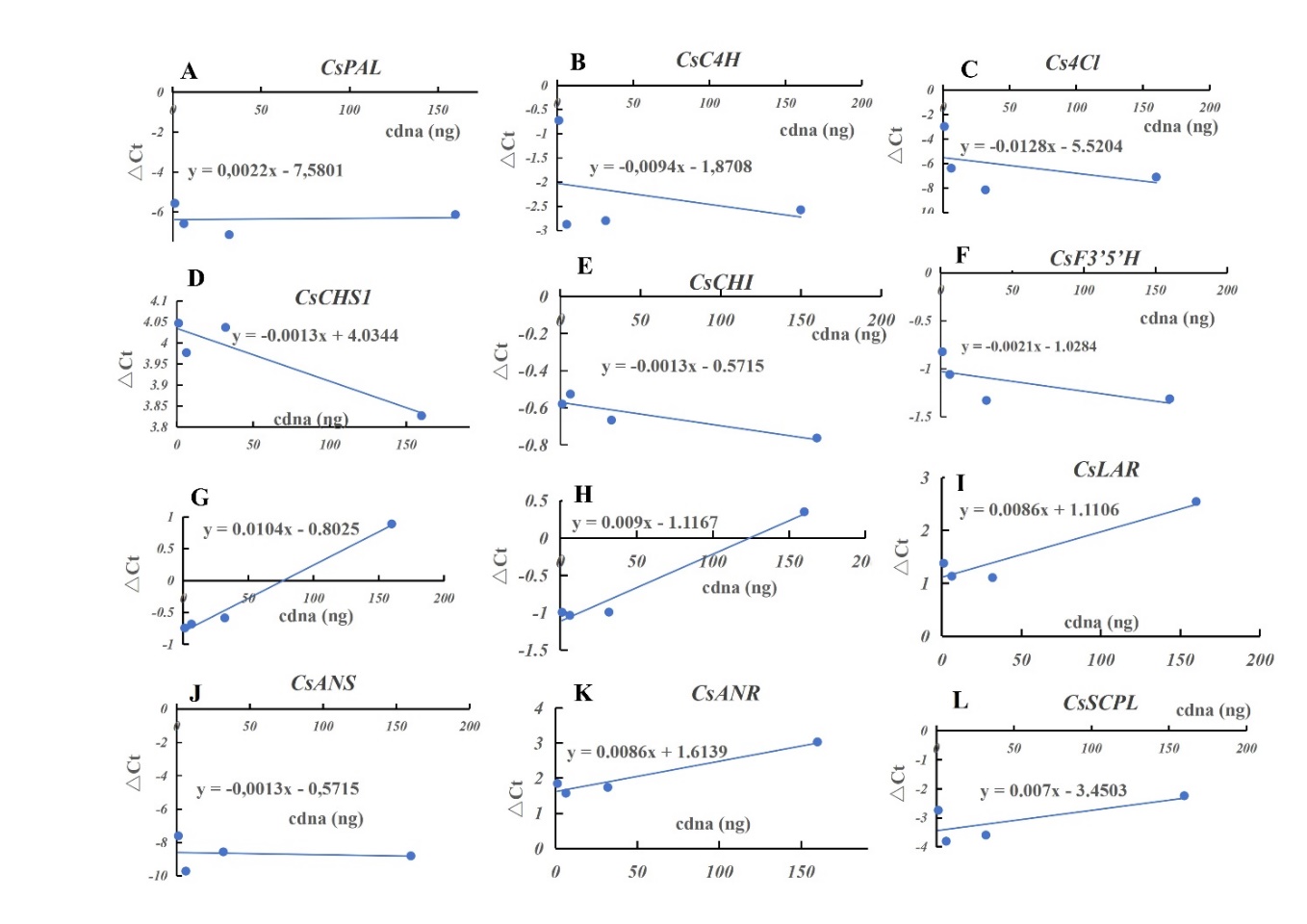


**Supplementary Figure 4.** Standard curves of amplification efficiency.

**
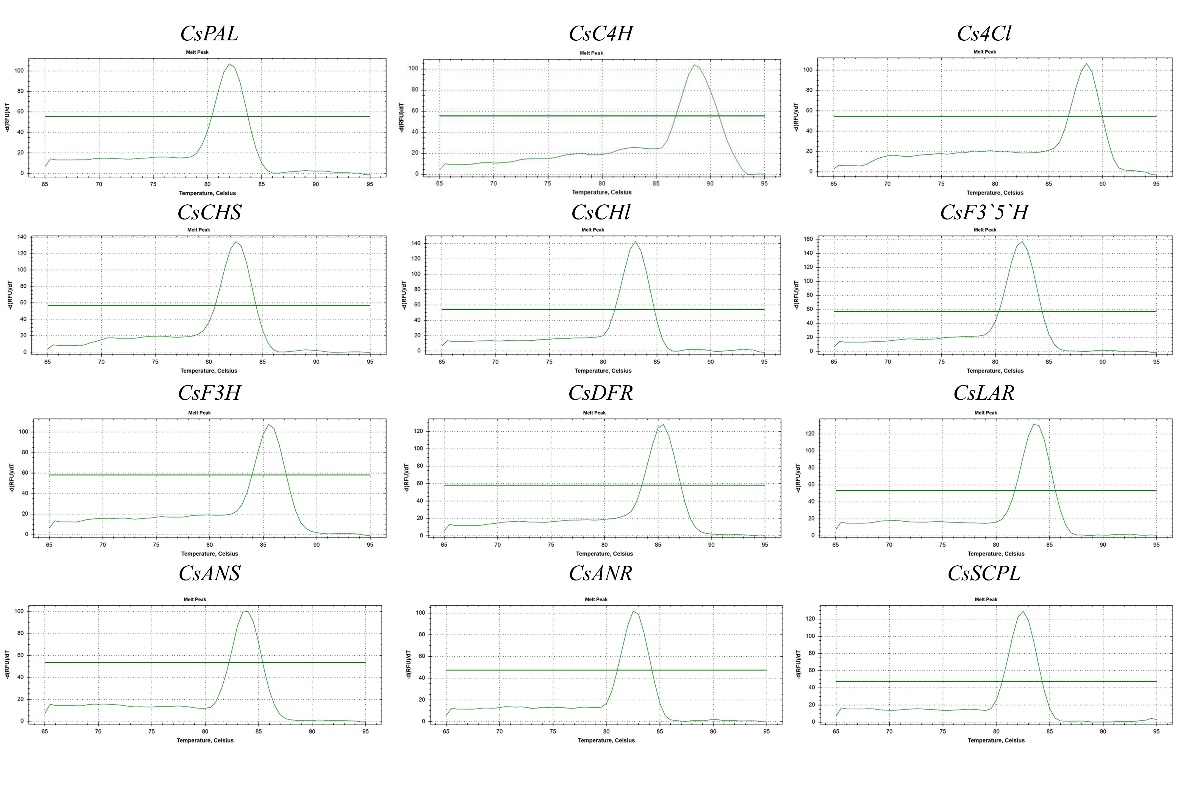
**

**Supplementary Figure 5.** Melting curve chart of gene qPCR experiment.


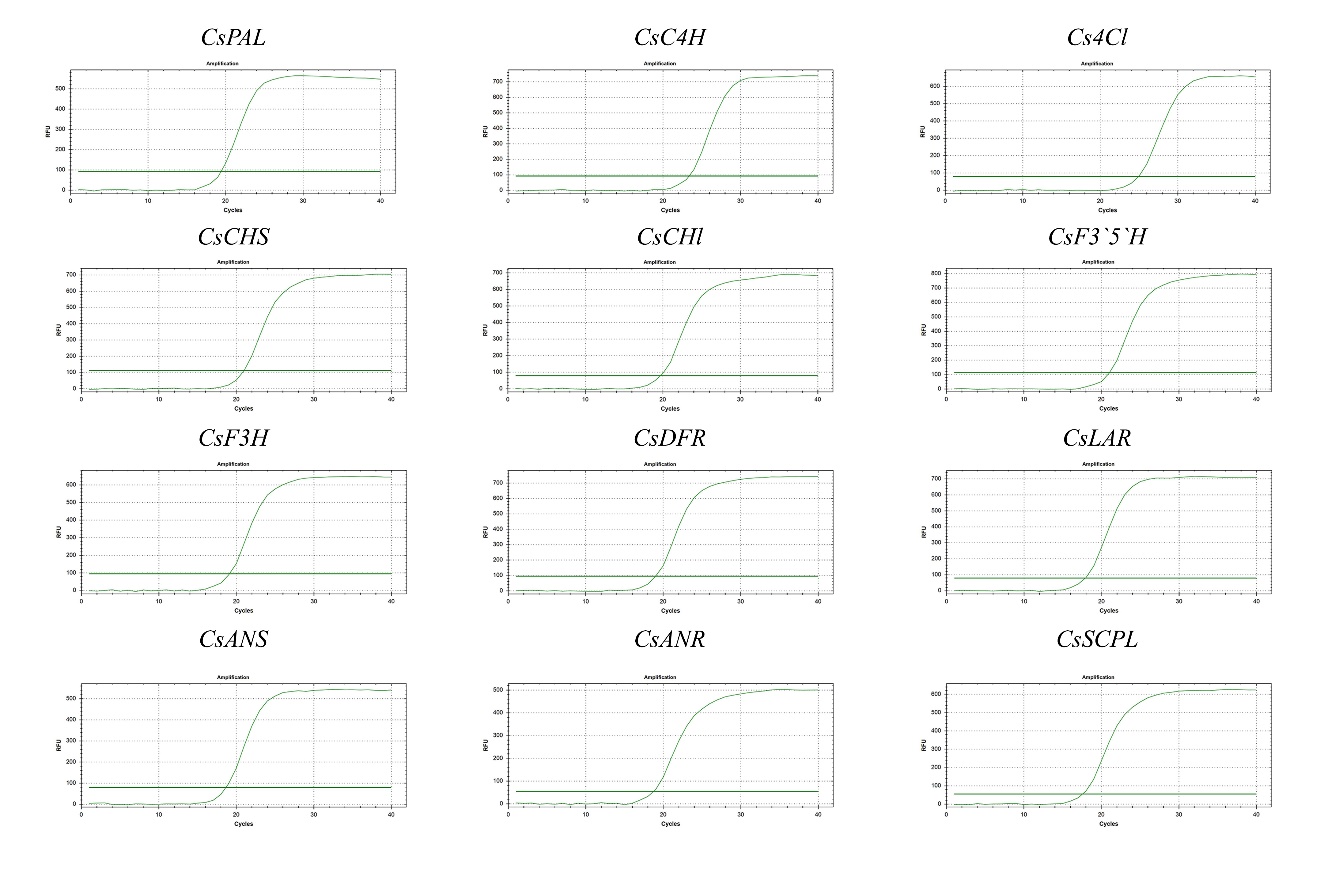
 **Supplementary Figure 6.** Amplification curve chart of gene qPCR experiment.
